# Rethinking avian influenza control: a systems-oriented One Health perspective for family poultry

**DOI:** 10.3389/fvets.2026.1814751

**Published:** 2026-10-19

**Authors:** Robyn Alders, Brigitte Bagnol, Md. Ahasanul Hoque

**Affiliations:** 1 Development Policy Centre, Crawford School of Public Policy, Australian National University, Canberra, ACT, Australia; 2 Socio-economic Working Group, OFFLU, WOAH-FAO Animal Influenza Network, Paris, France; 3 Cummings School of Veterinary Medicine, Tufts University, North Grafton, MA, United States; 4 One Health Institute, Chattogram Veterinary and Animal Sciences University, Chattogram, Bangladesh

**Keywords:** community engagement, gender, multi-hazard risk management, One Health, systems approach, vaccine-preventable poultry diseases, avian influenza

## Abstract

Early detection and control of high- and low-pathogenicity avian influenza (HPAI, LPAI) in family poultry flocks remain challenging in settings where mortality from enzootic diseases such as Newcastle disease (ND) and infectious bursal disease (IBD) is persistently high. High background mortality obscures recognition of unusual disease events and delays reporting. In addition to epidemiological constraints, rebuilding trust among family poultry producers, particularly those affected by uncompensated culling during previous HPAI outbreaks, is essential for effective control. Locally grounded, participatory approaches that recognize the lived experience of both women and men poultry keepers are required to strengthen surveillance, reporting, and response. This policy-oriented rapid review synthesized 110 peer-reviewed publications and 27 policy and guidance documents to examine the structural, epidemiological and socio-economic factors influencing HPAI and LPAI prevention and control in family poultry systems. Effective HPAI and LPAI control should be embedded within a broader, systems-oriented strategy that improves overall poultry productivity and explicitly values smallholder knowledge and participation. A multi-hazard risk management approach grounded in One Health is required, i.e., one that addresses equity, food and nutrition security, livelihoods, climate resilience, and global health. Building on a nine-factor assessment of the factors shaping avian influenza control feasibility, we group practical entry points into three complementary themes: (1) structural constraints within family poultry production systems, including the use of locally adapted poultry genotypes, safe, improved access to safe markets and sustainable feed systems, and effective control of enzootic vaccine-preventable diseases; (2) governance, institutional and social barriers, including culturally appropriate outbreak preparedness and response protocols; (3) gender and socio-economic equity, encompassing participatory approaches grounded in inclusivity, transparency, and accountability. Functional cross-sectoral collaboration between public health, animal health, and environmental sectors, along with local government, is essential if One Health approaches are to deliver tangible, cost-effective reductions in zoonotic risk and effective response while supporting sustainable household food and nutrition security.

## Introduction

Poultry domestication dates back several thousand years and represents one of the earliest and most widespread forms of animal husbandry (1). Until the mid-twentieth century, poultry were typically raised in small flocks by rural and peri-urban households using locally available resources and family labor (2, 3). Today, family poultry systems continue to predominate across many low- and middle-income countries (LMIC), fulfilling critical roles in livelihoods, food and nutrition security, equity, and cultural practice (2, 4, 5).

Over recent decades, however, the global poultry sector has undergone profound structural change. The rapid expansion of highly intensive, commercial poultry production has reshaped production systems, value chains, and regulatory frameworks, particularly in export-oriented economies (6). Animal health and biosecurity policies have largely been developed through engagement with large-scale commercial actors, often with insufficient consideration of the realities of small-scale and family poultry producers (7, 8). This policy mismatch undermines both the relevance and uptake of avian influenza control measures in small- and medium-scale poultry systems (9).

### Characteristics of family poultry

The FAO defines family poultry as production systems that rely primarily on family labor and locally available inputs and may include chickens, ducks, geese, pigeons, quail, and guinea fowl (10). Three broad production categories are commonly recognized: extensive (i.e., free-range) or scavenging systems (approximately 1–50 birds); semi-intensive systems (approximately 51–200 birds); and small-scale intensive systems (generally >200 broilers or >100 layers) (10). Each system requires biosecurity measures that are appropriate to the production context (10). In many low- and middle-income settings, these family poultry systems coexist within the same community, necessitating cooperative, village-level approaches to biosecurity, particularly where birds roam freely and the village effectively functions as the epidemiological unit. Extensive and semi-intensive family poultry systems also make an important contribution to sustainable food systems by enabling birds to obtain a substantial proportion of their diet through scavenging, crop residues, insects, household food scraps and locally available feed resources. Consequently, these systems generally reduce direct competition between poultry and people for human-edible grains and other feed ingredients compared with more intensive production systems, while converting otherwise underutilized biomass into nutrient-rich food (11, 12). Effective biosecurity must also extend beyond individual households to encompass the entire poultry value chain, including live bird markets and transport networks for inputs and outputs; however, implementation remains challenging, especially for resource-constrained small-scale intensive producers with narrow profit margins and volatile feed costs. Because family poultry is commonly kept in close proximity to human dwellings, inadequate biosecurity can increase opportunities for zoonotic disease transmission (10). As production intensity increases, dependence on externally sourced feed, replacement stock, veterinary inputs and equipment also increases, elevating the risk of pathogen introduction through movements of feed, chicks, personnel, vehicles and equipment and increasing the consequences of biosecurity failures (13–16).

In many contexts, poultry are primarily managed by women, who exercise day-to-day responsibility for feeding, health care, and marketing (5, 17, 18).

### Scope of this contribution

This policy-oriented rapid review aims to (i) examine the historical, structural, and socio-economic factors that have contributed to the emergence, spread, and persistence of HPAI and LPAI in LMIC; and (ii) identify practical, systems-oriented interventions for their prevention and control. These interventions are synthesized into a conceptual systems framework that integrates One Health principles with a multi-hazard risk management approach to infectious poultry disease control while contributing to food security, livelihoods, climate resilience, and global health.

## Methods

This policy-oriented rapid review aims to synthesize current evidence on avian influenza control in family poultry systems within a One Health, multi-hazard risk management framework. Rapid reviews streamline elements of systematic review methodology to provide timely, policy-relevant evidence for complex, practice-oriented questions (19).

The literature search strategy, databases searched, search strings, and study selection summary are provided in Supplementary Table S1. Searches used predefined combinations of terms relating to avian influenza, family poultry production systems, One Health and multi-hazard risk reduction. Table 1 summarizes the identification, screening and selection of peer-reviewed literature retrieved from PubMed and Web of Science, together with the policy and guidance documents included to provide contextual and normative perspectives. Titles and abstracts were screened for relevance, followed by full-text review of eligible publications. Publications were screened by the lead author and considered eligible when they addressed one or more aspects of avian influenza (HPAI or LPAI) relevant to family poultry production systems, particularly in low- and middle-income countries, including epidemiology, disease emergence or transmission, surveillance, biosecurity, vaccination, veterinary services, poultry production and value chains, risk communication, governance, community participation, gender, socio-economic or cultural factors, or approaches relevant to One Health and multi-hazard risk management. Studies addressing related poultry diseases were also considered where they provided relevant evidence on disease recognition, background mortality, vaccination, surveillance or control of avian influenza. Policy, technical and guidance documents were included where they provided authoritative contextual, normative or operational information relevant to avian influenza prevention and control, family poultry systems, One Health or multi-hazard risk management. Publications were considered irrelevant and excluded when they had no substantive connection to avian influenza control or its relevant determinants in family poultry systems, addressed unrelated animal or human health issues, focused exclusively on production systems or diseases without relevance to avian influenza control, or provided information that was not relevant to the review objectives. Screening covered all publicly available online references up to 20 July 2026. Additional policy documents and technical reports were identified through targeted searches of FAO, WOAH, WHO and UNDRR websites up to 10 August 2026. Study screening, eligibility assessment and data extraction were undertaken by one author to meet the timeframe and pragmatic requirements of a rapid review.

**Table 1:** Summary of the literature search, study selection and inclusion of policy and guidance documents.

Review stage	Number (*n*)
Records identified through database searching	1,093
Duplicate records removed	236
Records screened and excluded by title only	392
Records screened by title and abstract	465
Records excluded	237
Full-text articles reviewed	228
Full-text articles excluded	118
Peer-reviewed publications included	110
Policy and guidance documents identified through supplementary searching	27
Total references included	137

Consistent with rapid review methodology, priority was given to high-quality, authoritative sources, including peer-reviewed reviews, key empirical studies, and technical reports. The aim was to identify major concepts, frameworks, and evidence trends rather than achieve exhaustive coverage.

Data were synthesized thematically, focusing on three themes: (1) structural drivers and system constraints; (2) governance dimensions; and (3) gender and socio-economic equity. Findings were interpreted using a systems-oriented perspective to identify context-relevant strategies for strengthening prevention and control. Themes were derived through iterative synthesis of the literature to identify recurring concepts, policy issues, and implementation challenges relevant to the prevention and control of HPAI and LPAI in family poultry production systems. The analysis included evidence relating to avian influenza epidemiology, poultry production systems, veterinary service delivery, surveillance, biosecurity, vaccination, communication, gender, socio-economic and cultural factors, governance, and One Health and multi-hazard risk management approaches, with findings integrated to inform the development of a conceptual framework and policy recommendations.

## Review findings and synthesis

The review findings are synthesized thematically below, beginning with the historical and epidemiological context of avian influenza, followed by consideration of poultry production systems, the burden of enzootic diseases, and the institutional and socio-economic factors influencing disease control.

### A brief history of avian influenza

Avian influenza is caused by bird-adapted influenza A viruses and was first formally described in Italy in 1878. Until the mid-twentieth century, HPAI was commonly referred to as “fowl plague,” a term that historically encompassed what are now recognized as distinct diseases, including fowl cholera (20) and Newcastle disease (ND) (21, 22). Avian influenza viruses (AIVs) naturally circulate among birds, particularly wild aquatic species such as ducks, geese, and shorebirds. Based on their pathogenicity in poultry under standardized laboratory conditions, AIVs are classified as LPAI or HPAI viruses (23, 24).

### The rise of HPAI

Between 1990 and 2023, the global human population grew from approximately 5.3 billion to 8.1 billion (25). Over a similar period, the global chicken population expanded from roughly 11 billion to more than 33 billion birds (26). This rapid growth in poultry numbers, particularly within high-density production systems (rather than extensive and semi-intensive systems), has created ecological and epidemiological conditions conducive to the emergence, re-emergence and global dissemination of HPAI viruses (15, 27).

Historically, AIVs circulating in wild birds were predominantly of low pathogenicity. As of July 2026, chickens, turkeys, and ostriches are the only species in which LPAI viruses have been known to evolve into highly pathogenic forms following sustained circulation in domestic poultry populations (28). Major avian influenza outbreaks in Mexico (1993–1995), Pakistan (1994–1995), and Italy (1999–2000) marked a turning point in global avian influenza epidemiology and control by demonstrating the capacity of H5 and H7 low-pathogenicity viruses to evolve into highly pathogenic forms in commercial poultry, prompting major advances in surveillance, vaccination, control and eradication policies (29–31).

Over the past eight decades, poultry production systems have become increasingly complex. Intensification has increased stocking densities, reduced genetic diversity, and accelerated movement of birds, feed, and equipment, amplifying the risk of disease transmission (32, 33). The emergence of HPAI H5N1 in southern China in 1996 (A/goose/Guangdong/1/1996) led to recurrent outbreaks across Asia, particularly in settings where ND and fowl cholera were already enzootic and poorly controlled (21). Subsequent spread has been driven by multiple interacting factors, including uncompensated mass culling that eroded producer trust (22, 34, 35); inappropriate carcass disposal leading to environmental contamination (36); and the widespread use of commercial chicken hybrids that tend to encounter a range of health issues when raised under resource-constrained small-scale intensive production systems (37–40). In many LMIC, limited access to formal poultry health services leads family poultry producers to rely on informal poultry health practitioners and drug vendors. This reliance can contribute to inappropriate or off-label antimicrobial use and reduce opportunities for timely reporting of suspected disease events through official veterinary surveillance systems (41).

The evidence base linking intensive poultry production systems with HPAI emergence and spread is heterogeneous. Well-supported evidence indicates that increasing poultry density, extensive poultry movement networks, and live bird market connectivity can facilitate the amplification and dissemination of AIVs once introduced into poultry populations (27, 33, 42–44). More broadly, the emergence of highly pathogenic influenza viruses, including H5N1 and other subtypes, has been associated with intensive poultry production systems (27). This is consistent with evolutionary theory, which suggests that high densities of susceptible hosts can favor the emergence and spread of more virulent pathogens (45). There is also strong evidence that inadequately managed carcass disposal, delayed detection, and uncompensated culling can discourage disease reporting and contribute to virus persistence (34–36, 46, 47). In contrast, several frequently cited drivers remain context-dependent mechanisms rather than universally established causal factors. Reduced genetic diversity and the predominance of high-production genetic lines may influence susceptibility, resilience, disease detection, or management practices under some circumstances, although their effects can be mediated by husbandry, biosecurity, nutrition, and broader production system characteristics (14, 15, 37, 38). The proposition that these factors interact synergistically to drive HPAI emergence, persistence, or evolution remains plausible and biologically credible, although direct empirical evidence demonstrating their combined contribution across diverse settings is limited (14, 15, 27, 37, 38). Further interdisciplinary research is therefore required to clarify the relative importance of these interacting factors and the conditions under which they may increase HPAI risk (14, 15, 27).

In relation to direct HPAI impacts on human health, poultry outbreaks in Hong Kong in 1997 resulted in the first recognized transmission of HPAI H5N1 to humans, with 18 laboratory-confirmed infections (48). Since 2003, more than 1,000 laboratory confirmed human H5N1 infections have been reported from over 20 countries, with the vast majority associated with direct or indirect exposure to infected poultry or contaminated environments (49–51). Most reported infections have occurred in LMIC among people keeping or handling family poultry, participating in household poultry slaughter and processing, or working in live bird markets. By contrast, relatively few confirmed cases have been linked to large-scale poultry production. During the ongoing clade 2.3.4.4b panzootic, however, the range of occupational exposure settings has expanded. In the United States, 70 confirmed human H5N1 infections were identified between March 2024 and May 2025, including 41 associated with infected dairy cattle, 24 associated with commercial poultry operations, two linked to family poultry, and three with no identified exposure source (52). These findings indicate that while household poultry exposure remains the principal source of zoonotic infection globally, intensive animal production systems can also present a risk when HPAI viruses become established in susceptible mammalian populations. Serological studies further suggest that subclinical and mild infections occur more frequently than indicated by routine surveillance, implying that laboratory-confirmed cases underestimate the true incidence of zoonotic H5N1 infection (48).

As of June 2026, the HPAI H5N1 clade 2.3.4.4b had reached every continent, causing unprecedented mortality in wild birds and infecting new host species, including dairy cattle in the United States (53–56).

### The emergence of zoonotic LPAI

Most influenza A subtypes identified in birds are LPAI viruses, reflecting their long-standing evolutionary association with wild avian hosts. Surveillance studies conducted from the mid-twentieth century onwards demonstrated that wild birds frequently carry influenza viruses without clinical disease (57). Subsequent monitoring confirmed that LPAI viruses constitute a genetically diverse reservoir that continually seeds domestic poultry populations.

Transmission of LPAI viruses from wild birds to domestic poultry occurs through direct and indirect contact, including shared water sources and contaminated environments (58). Such transmission events are more likely in production systems where poultry have access to open environments and interact with wild birds, including scavenging and free-range systems (42, 59).

Within domestic poultry populations, LPAI viruses can circulate with limited or no overt clinical signs, particularly in chickens, ducks, geese and turkeys, making detection through passive surveillance challenging (23, 60). Despite their generally low pathogenicity, LPAI infections have been associated with reductions in egg production, growth performance, and increased susceptibility to secondary infections (61, 62). While HPAI surveillance frequently relies on passive reporting of unusual mortality events, effective LPAI surveillance generally requires active sampling because infected birds may show few or no clinical signs (20, 23, 28).

Of particular importance, LPAI viruses of the H5 and H7 subtypes have been shown to mutate to become highly pathogenic forms following sustained circulation in poultry populations (23, 28). This process has been documented in multiple outbreaks and underpins the global emphasis on early detection and control of these subtypes in domestic birds (28).

Live bird markets are recognized as important environments for the maintenance and transmission of AIVs, as they aggregate birds from multiple sources and facilitate viral spread through high-density contact networks (42, 43). Modelling and field studies have demonstrated that such market systems can sustain viral circulation and contribute to onward transmission within poultry value chains (43, 44).

In addition to poultry trade networks, mixed-species production systems, particularly those involving ducks and chickens, contribute to the persistence and evolution of LPAI viruses due to differences in host susceptibility and viral shedding dynamics (58, 61). Ducks, in particular, may excrete virus without showing clinical signs, facilitating silent transmission (58).

Seasonal patterns in LPAI transmission have been associated with wild bird migration and ecological factors that influence contact between wild and domestic bird populations (58). These dynamics contribute to the continual introduction of genetically diverse viruses into poultry production systems (58). Although most documented human influenza A infections involve HPAI viruses, several LPAI viruses have also demonstrated zoonotic potential, most notably H9N2 and H7 subtypes, typically resulting in mild disease, although severe cases have been reported. These infections occur primarily among individuals with close contact with infected poultry (24, 61, 63, 64). H9N2 viruses have caused sporadic human infections since the late 1990s, primarily in Asia and the Middle East, while human infections with LPAI H7N1, H7N3, and H7N7 have also been reported, mainly among poultry workers during outbreaks (64).

### Understanding the disease and the host

#### Characteristics of HPAI and factors influencing control

HPAI viruses can persist in the environment for weeks or months, particularly at low temperatures and high moisture levels. Once introduced into poultry systems, high stocking densities and frequent movement of birds, people, and equipment facilitate rapid transmission within and between farms (65). Early control strategies relied heavily on mass depopulation, an approach that proved socially, economically, and ethically problematic in many LMIC settings (34, 46, 47).

Since the mid-1990s, increasing numbers of countries have incorporated inactivated vaccines into HPAI control strategies. However, vaccination remains challenging to implement effectively at scale, especially where veterinary services, cold chains, and monitoring systems are weak (66, 67).

#### Characteristics of LPAI and factors influencing control

In wild birds, LPAI viruses maintain influenza A virus diversity and enable genetic reassortment, contributing to the emergence of novel viruses (58). Like HPAI viruses, LPAI viruses can persist in the environment for extended periods under favorable conditions.

Surveillance for LPAI in many low- and middle-income countries remains limited because of constraints in funding, laboratory capacity and veterinary infrastructure, with surveillance efforts frequently prioritized towards high pathogenicity avian influenza owing to its greater impacts on poultry mortality, public health and international trade (9, 23, 68). The subclinical nature of LPAI infections in poultry complicates detection through passive surveillance systems, necessitating active surveillance approaches where feasible (23). Given that live bird markets and poultry trade networks have been identified as key sites of virus maintenance and transmission, targeted surveillance in these settings has been widely used to improve detection efficiency (42, 43). Unlike HPAI, routine vaccination against LPAI is rarely implemented in family poultry systems due to cost, logistical constraints, and the potential to mask viral circulation, complicating surveillance efforts (47, 66).

#### The poultry host and production systems

Global poultry production systems range from highly intensive commercial operations to small-scale, backyard and informal market-based systems, each presenting distinct vulnerabilities to LPAI and HPAI infection (10, 23).

Intensive commercial systems generally apply formal biosecurity measures designed to reduce virus introduction from wild birds and external contacts (42). However, once infection occurs, high stocking densities and frequent movements of birds, people and equipment can facilitate rapid within-farm and between-farm transmission of both LPAI and HPAI viruses (65).

Contrastingly, small-scale, family poultry production systems, which dominate in many low- and lower-middle-income countries, often have limited biosecurity and allow free ranging, mixing of species and frequent contact with wild birds, increasing the likelihood of virus introduction and persistence (10, 59, 69). Mixed-species rearing, particularly involving ducks and chickens, increases opportunities for viral reassortment and persistence of LPAI viruses due to differences in susceptibility and shedding dynamics (58, 61). These systems are commonly linked to informal poultry value chains, where disease surveillance and regulatory oversight are weak (43). Nevertheless, scavenging systems demonstrate resilience and adaptability and require biosecurity measures tailored to local realities rather than replication of industrial models (59).

Live bird markets play a central role in poultry value chains in many regions and are recognized as key nodes for avian influenza transmission, as they aggregate birds from multiple sources and enable viral amplification and onward spread (44). The structure and governance of poultry input and output value chains, therefore, strongly influence the risk of avian influenza transmission and persistence, with poorly regulated and fragmented chains associated with greater vulnerability to both LPAI and HPAI transmission (70).

#### The burden of enzootic diseases

LPAI infections in poultry are often subclinical or mild. Despite often mild clinical presentation, LPAI infections can reduce overall flock productivity, particularly when combined with other endemic infections, thereby imposing cumulative economic losses on smallholder households (61, 62).

In contrast, the clinical signs of HPAI in chickens are indistinguishable from those of ND, fowl cholera, and IBD, complicating field diagnosis without laboratory confirmation (60). ND, first described in 1926, remains enzootic across much of Africa, Asia and South America (71, 72).

In family poultry systems, vaccination against ND and other key vaccine-preventable diseases, is often irregular or absent (10, 73, 74). This results in frequent high-mortality events that normalize poultry deaths and diminish the likelihood of HPAI being reported or even recognized (34, 75). Coping strategies that follow ND outbreaks, such as the sale of live or dying birds, home slaughter and consumption, or improper disposal, can exacerbate transmission and zoonotic risks when HPAI is circulating (76).

### Challenges in controlling HPAI and LPAI in family poultry production systems

HPAI and LPAI control strategies developed for medium- and large-scale commercial operations often falter in family poultry settings. This mismatch reflects fundamental differences in scale, resource availability, production objectives, risk assessment, and socio-cultural contexts. Nine key factors that have been reported to influence the outcome of HPAI control activities are outlined in Table 2 [derived and adapted from (73)], with each factor described in more detail in the following section.

1 Trust in political and veterinary leadership

**Table 2:** A simplified heuristic framework summarizing nine factors that shape AIV epidemiology and affect the feasibility and effectiveness of HPAI control, organized according to the domains depicted in Figure 1.

	Factors influencing HPAI control feasibility	HPAI control easier	HPAI control more difficult	Supporting references
Governance and institutional capacity				
1	Trust in political and veterinary leadership	Higher	Lower	(77–79, 113, 135)
2	Links between veterinary services and producers	Better resourced	Poorly resourced	(6, 42, 62, 71, 73, 80–85, 125, 136)
3	Veterinary services’ capacity to integrate local knowledge systems	Higher	Lower	(71, 73, 86–89, 137)
Value chains and markets				
4	Poultry value chain quality assurance activities	Stronger	Weaker	(6, 43, 76, 90, 91)
Community, participation and trust				
5	Percentage of households raising poultry	Lower	Higher	(42, 73, 113)
6	Involvement of family poultry producers in technical solution development	Higher	Lower	(5, 42, 92–94, 102, 103)
Surveillance, communication and preparedness				
7	Veterinary services develop communication messages with farmers	Higher	Lower	(42, 92, 95–97)
Core systemic risk				
8	Annual poultry mortality	Lower	Higher	(34, 42, 92, 99, 106)
Cross-cutting: gender equity				
9	Gender awareness	Higher	Lower	(17, 100–105)

Derived and adapted from (73).

The capacity of veterinary services to foster respect, empathy, and knowledge, and to respond to small-scale farmers’ specific needs contributes to the development of trust (77, 78). Trust in political and veterinary leadership increases the effectiveness of pandemic control because public health and animal health strategies rely on voluntary compliance, accurate information sharing, and community cooperation. When citizens question the competence or intentions of authorities, adherence to recommended biosecurity and reporting measures declines (79).

2 Links between veterinary services and producers

In many rural settings, veterinary services are under-resourced or absent, with deficiencies most pronounced in underserved communities (42, 80, 81). Veterinary services are often oriented toward large livestock (e.g., cattle, camels, goats and sheep) and commercial poultry enterprises, with comparatively limited engagement in small-scale chicken and family poultry production systems (73, 82). As a consequence, veterinary services may lack an in-depth understanding of the constraints, priorities, and disease dynamics affecting the family poultry sub-sector. In addition, veterinary services may adopt a top-down approach trying to impose solutions developed for the large-scale sector without compensating small-scale farmers when their flocks are culled. This approach tends to widen the gap and negatively impact trust between small-scale farmers and veterinary services (83, 84).

Availability and access to effective poultry vaccines remain significant limitations in many low- and middle-income countries. Inadequate financing for vaccine procurement or local production, together with absent or unreliable cold-chain infrastructure, are persistent barriers (42, 62, 71). Evidence from several low- and middle-income settings indicates that poultry are frequently assigned lower priority for vaccination and animal health investments than larger livestock species (71, 73, 82). Reports from Bangladesh and Guinea suggest that improved cross-sectoral collaboration between animal and public health authorities can strengthen vaccine delivery systems, including cold-chain training, maintenance, and joint monitoring of vaccination coverage (85).

3 Veterinary services’ capacity to integrate local knowledge systems

Livestock keepers may draw on plural explanatory models of animal disease, including biomedical, ethnoveterinary, spiritual and culturally embedded interpretations (73, 86–88). These understandings may influence how disease signs are interpreted and how interventions such as vaccination, disinfection or depopulation are perceived and acted upon (73, 86). For example, studies in East, Southern, and West Africa show that livestock keepers may consult traditional healers or use herbal remedies before contacting formal veterinary services (71). In such contexts, veterinary interventions are more likely to be accepted when integrated with local knowledge systems and veterinarians engage with local people seen as healers and knowledge holders (89).

4 Poultry value chain quality assurance activities

Quality assurance systems are essential for delivering safe food and reliable animal health services (90). In many resource-limited settings, poultry that are sick or have died from disease are consumed within households or sold at reduced prices to neighbors or nearby restaurants and markets, increasing public health risks (76, 91). During HPAI outbreaks, market interventions primarily aim to interrupt explosive transmission, whereas for LPAI they focus on reducing long-term viral maintenance and opportunities for reassortment (20, 22, 28, 43).

5 Percentage of households raising poultry

During the first wave of HPAI H5N1 (i.e., 2003–2008), countries where a high proportion of households raised poultry often faced greater institutional and financial challenges in engaging effectively with large numbers of small-scale producers across diverse geographic, linguistic and cultural contexts (42, 73). These constraints limited surveillance coverage, participatory engagement with family producers, risk communication, and timely response (7, 9, 22, 42, 73).

6 Involvement of family poultry producers in technical solution development

Limited involvement of family poultry producers, particularly women, in veterinary service-led initiatives leads to misaligned interventions, reduced uptake of innovations, mistrust, and economic losses. Participatory co-development approaches are associated with improved disease control outcomes and sector resilience (5, 92–94).

Financial constraints and risk perception limit the adoption of biosecurity measures designed for intensive systems, such as housing, fencing, and controlled feeding. Context-appropriate technical solutions should be co-designed to reduce interspecies mixing and contact with wild birds, and to promote farmers’ understanding of available alternatives and disease risks. Informal live bird markets, while economically and socially critical, require participatory redesign to reduce viral amplification and reassortment risks while ensuring buy-in of stakeholders, specifically women (5, 42).

7 Veterinary services develop communication messages with small-scale farmers

In the 21st century, risk communication continues to rely on one-way communication channels, including printed materials and radio or television announcements delivered in official national languages (95). In low-literacy settings, poultry producers, particularly women, often communicate primarily in local languages, rendering such materials ineffective (92, 96, 97).

Messages are often developed in isolation from producers and specialists familiar with family poultry systems. Poorly tailored communication has contributed to continued HPAI transmission in settings where large numbers of households keep small flocks (22, 89). Communication strategies should extend beyond mortality-based messaging to include productivity losses and long-term risks associated with LPAI circulation (62, 92). Low levels of formal education further limit the adoption of technological innovations (98) because communication materials are usually not developed together with the farmers. These challenges to engagement underscore the importance of building trust with local communities and leveraging communications via existing networks (e.g., faith leaders, women’s and youth groups) (42).

8 Annual poultry mortality

In many countries affected by rapid HPAI H5N1 spread, family poultry producers were accustomed to high baseline mortality due to uncontrolled vaccine-preventable diseases such as ND and IBD (34, 92). Even large commercial operations in tropical LMIC settings experience significant losses due to ND and IBD, despite vaccination (99).

Drivers of vaccination failure include vaccine thermostability challenges, strain mismatch, logistical and administration errors, and viral evolution (99). Such failures can mask the incursion of HPAI, delaying detection and response and contributing to farmers’ mistrust toward veterinary services.

9 Gender awareness

HPAI H5N1 response strategies have been hindered by a lack of gender integration at multiple levels (100, 101). Program planners and technicians, predominantly men, tend to focus narrowly on the technical aspects of prevention and control, without considering gender as a planning and analytical tool (17, 102). This is compounded by the absence of systematic gender analysis of HPAI H5N1 socio-economic and cultural impacts (100), the scarcity of gender-disaggregated data in research and monitoring, and the lack of gender policies in most implementing agencies. Moreover, weak gender awareness, limited programming capacity, and the underrepresentation of women and women-focused organizations in the HPAI H5N1 response and planning have further restricted the effectiveness and inclusivity of interventions (17). Gender equity is central to effective HPAI control. Women frequently manage family poultry flocks while facing limited access to vaccines, training, and decision-making forums, because family poultry are frequently de-prioritized within veterinary services and because gender norms and structural barriers often limit women’s engagement with formal poultry health services. These barriers include restricted control over financial resources, limited decision-making authority within households, competing domestic and caregiving responsibilities, constraints on mobility and travel to veterinary service providers, lower access to education and training opportunities, and livestock extension systems that predominantly engage with men or focus on larger commercial livestock enterprises. As a result, women frequently rely on informal poultry health providers, community networks, or their own experience when managing poultry health (103–105). Addressing gender-related barriers and opportunities across the poultry value chain, including improving women’s access to veterinary extension, training, producer organizations, and decision-making processes, may strengthen vaccine access, adoption, and delivery, thereby improving the effectiveness and equity of poultry health systems (18, 82, 83, 106).

### Toward a One Health, multi-hazard risk management approach

The global spread of HPAI demonstrates the inadequacy of siloed, single-hazard approaches to disease management. Contemporary risk assessment frameworks increasingly recognize that biological and environmental hazards interact with patterns of exposure, structural and socio-economic vulnerabilities, and institutional capacities to create cumulative, cascading and systemic risks (107, 108).

Multi-hazard risk management frameworks provide structured methodologies for assessing these interacting risks. Hochrainer-Stigler et al. (108) propose a six-step framework that examines risk across a continuum from single hazards to multi-hazard and systemic risk, recognizing that hazards generate cascading impacts across interconnected systems. Similarly, Shi et al. (109) describe an evidence-based, bottom-up framework for multi-scale, multi-hazard risk screening that evaluates exposure, vulnerability, criticality, asset risk, coping capacity and overall risk across administrative levels. Applied to avian influenza, these approaches can facilitate identification of geographical ‘hotspots’ where hazards, exposure pathways, vulnerabilities and capacity constraints converge, for example, viral circulation in wild birds, high poultry densities, extensive poultry movement networks, inadequate control of vaccine-preventable poultry diseases, weak surveillance and limited laboratory capacity.

While multi-hazard frameworks explain how multiple risks interact, a One Health approach expands this perspective by explicitly integrating human, animal and environmental health within coordinated governance systems. The One Health Joint Plan of Action (OH JPA) 2022–2026 (107) recognizes that zoonotic disease emergence and transmission are driven by interacting biological, environmental, ecological, climatic, social, economic and governance factors operating across the human–animal–plant–environment interface. Accordingly, it promotes integrated prevention, preparedness, detection, response and recovery measures that simultaneously address multiple risk drivers rather than focusing solely on pathogen control.

The global spread of HPAI H5N1, with extensive poultry losses and sporadic human infections and deaths, has reinforced the need for cross-sector collaboration among veterinary, public health and environmental sectors (31, 110, 111). One Health recognizes these sectors as interdependent and provides governance mechanisms to coordinate action across them (111). At the international level, FAO, WHO and WOAH, later joined by UNEP, formalized this collaboration through the Quadripartite One Health Joint Plan of Action (107). More broadly, One Health and Planetary Health approaches support integrated action that simultaneously promotes human, animal and environmental health while contributing to food and nutrition security, livelihoods, climate resilience, biodiversity conservation, gender equality and health equity (112, 118). They also encourage collaboration among multiple sectors, disciplines and communities and incorporate benefit–cost considerations into national disease control strategies, supporting more sustainable poultry health programs (113–118).

Drawing together the literature reviewed above, we consider the complementary strengths of multi-hazard risk management and One Health to be particularly relevant to HPAI and LPAI in family poultry systems. Family poultry production operates within highly interconnected biological, environmental and socio-cultural systems where disease emergence and control are shaped not only by pathogens but also by livelihoods, food security, gender, governance and local knowledge (5, 10, 59). In many low- and middle-income countries, family poultry contributes substantially to household nutrition, income and resilience, particularly among marginalized populations (2, 4, 5, 82). Consequently, biosecurity measures or culling programs implemented without consideration of these broader social and economic contexts may undermine trust in veterinary services, discourage disease reporting and reduce community participation in disease control (7, 35, 79).

Based on the evidence reviewed, we propose that biological hazards should be analyzed together with the exposure pathways, socio-economic vulnerabilities and institutional and community capacities that shape their likelihood and consequences. These include livelihood vulnerability, food insecurity, culturally embedded poultry management practices, social cohesion, dependence on informal markets and access to animal health services (5, 10, 114, 118, 119). Economic pressures that discourage disease reporting, dependence on informal poultry markets, limited access to finance for biosecurity improvements and the integration of poultry into household and community life all influence outbreak risk and response effectiveness (2, 3, 35, 59, 70). FAO (111) similarly emphasizes that effective avian influenza management requires coordinated action across livestock production, wildlife conservation and public health while safeguarding livelihoods and food security. Failure to embed prevention strategies within culturally appropriate and economically feasible frameworks may undermine both sustainability and equity, disproportionately transferring the costs of disease control to resource-poor households (7, 59, 92, 93).

Building on the literature reviewed above, we propose an integrated conceptual systems framework for avian influenza control in family poultry production that combines multi-hazard risk management with One Health principles (107, 111). In our interpretation, avian influenza risk emerges through interactions among biological and ecological hazards, exposure pathways, underlying vulnerabilities, and response capacities. Biological and ecological hazards include viral circulation, mutation and spillover and interactions between wild and domestic birds. Exposure is shaped by poultry density, bird movements, market connectivity and biosecurity conditions. Vulnerability is influenced by livelihood dependence on poultry, food insecurity, poverty and gendered inequalities in access to resources and services. Response capacity and resilience depends on surveillance and diagnostic capacity, veterinary services, institutional coordination, community trust and the ability to implement appropriate disease-control measures. These dimensions interact dynamically: for example, livelihood dependence may reduce willingness to report or cull infected flocks, while weak biosecurity and limited diagnostic capacity can further amplify transmission risk (108).

Our synthesis further suggests that although both HPAI and LPAI control benefit from this integrated perspective, the operational emphasis differs. For HPAI, One Health supports coordinated emergency outbreak management across veterinary, public health and environmental sectors. For LPAI, it places greater emphasis on long-term ecological surveillance, wildlife monitoring, prevention and understanding viral evolution (23, 28, 106, 107, 111). Together, multi-hazard risk management and One Health move beyond reactive disease control towards anticipatory, systems-based resilience by addressing not only outbreaks themselves but also the interacting biological, environmental and socio-economic conditions that enable disease emergence while respecting the livelihoods and cultures that sustain family poultry production.

Figure 1 presents the authors’ conceptual systems framework, developed through synthesis of the literature reviewed in this paper. Rather than depicting a single published model, it integrates evidence from One Health, multi-hazard risk management, family poultry production, participatory epidemiology and health systems research into a single conceptual representation. The figure incorporates the nine factors presented in Table 2 by embedding them within this broader framework as interconnected determinants that influence disease emergence, surveillance, prevention, response and the effectiveness of control measures across family poultry production systems.

**Figure 1:**
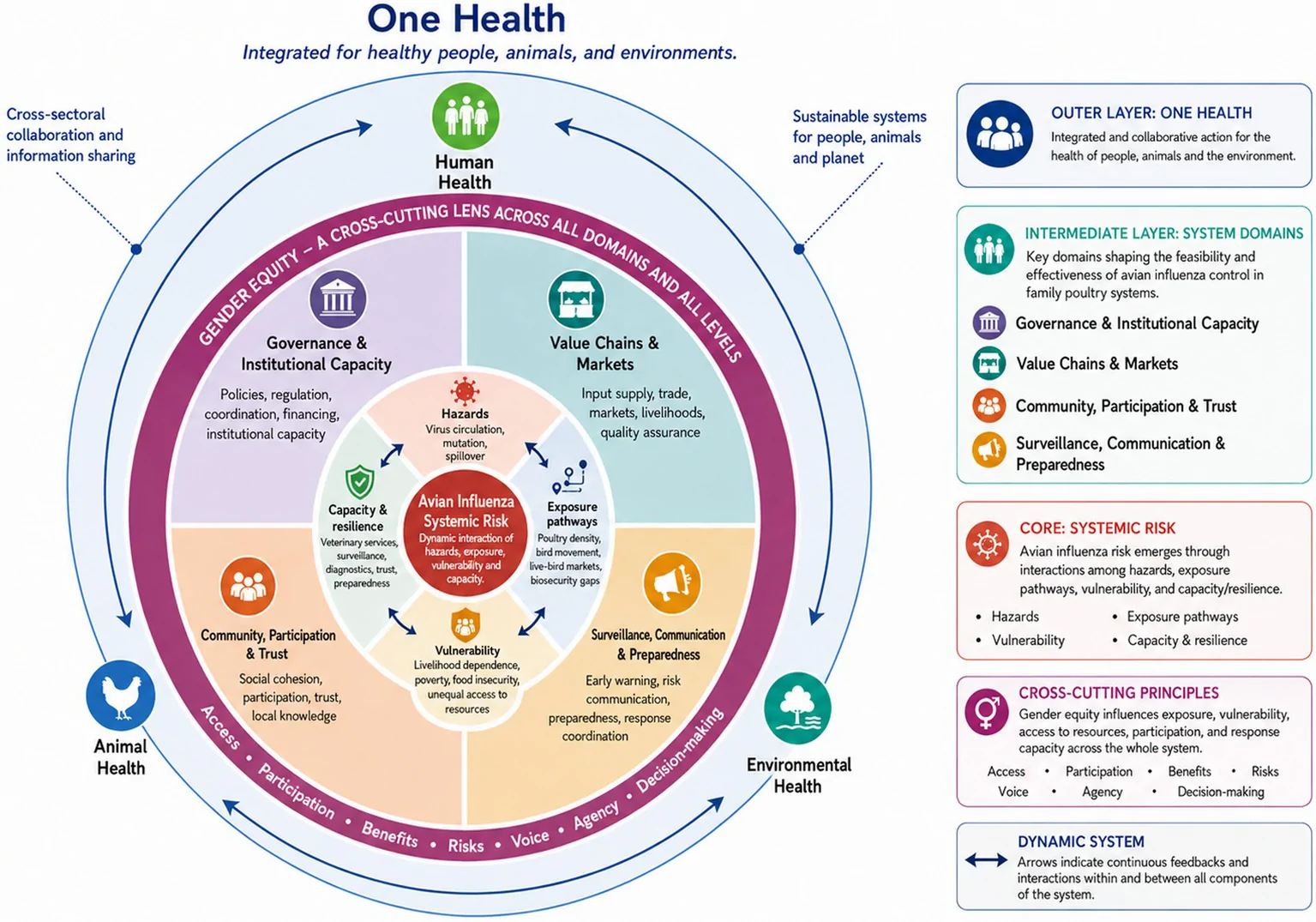
A conceptual systems framework for avian influenza control in family poultry systems integrating One Health principles with a multi-hazard risk management approach. Outer layer – One Health: integrated and collaborative action for the health of people, animals and the environment. Cross-cutting principles – Gender equity and other equity considerations: gender and other social inequalities influence exposure, vulnerability, access to resources and services, participation, benefits, risks, voice, agency, decision-making, and response capacity across the system. Intermediate layer – System domains: governance and institutional capacity; value chains and markets; community, participation and trust; and surveillance, communication and preparedness. Core – Systemic risk: avian influenza risk emerges through dynamic interactions among biological and ecological hazards, exposure pathways, underlying vulnerabilities, and capacities and resilience. Dynamic system: arrows indicate continuous feedbacks and interactions within and between components of the system. The framework represents the authors’ conceptual synthesis of evidence drawn from multiple published sources.

### Recommendations for family poultry production systems

The recommendations below are organized according to the principal structural, institutional and socio-economic drivers identified in this review as contributing to the persistence and spread of HPAI and LPAI in family poultry systems. Each recommendation addresses one or more of the nine factors summarized in Table 2 and is mapped accordingly in Table 3.

A Addressing structural constraints within family poultry production systems

1 Prioritize control of enzootic vaccine-preventable diseases

**Table 3:** Alignment of the proposed recommendations with the factors outlined in Table 2, together with the authors’ qualitative assessment of their feasibility of implementation in resource-limited settings informed by the literature.

Recommendation	Table 2 factors addressed	Feasibility in resource-limited settings*	Main constraints	Potential enabling factors	Expected impact*	Supporting references
A1. Prioritize control of enzootic vaccine-preventable diseases	8	Moderate	Vaccine supply, cold-chain limitations, laboratory capacity, sustained funding, workforce requirements for participatory surveillance	Thermotolerant vaccines, community animal health workers, integration with existing ND programs	Very high	(2, 22, 34, 42, 54, 62, 69, 71, 73–75, 99, 106, 120, 137)
A2. Strengthen poultry value chain quality assurance	4	Low-moderate	Infrastructure costs, regulation of informal markets, trader compliance, fragmented governance	Incremental market improvements, trader engagement, local government support	High	(22, 42, 43, 70, 76, 90, 91, 125)
A3. Select locally adapted poultry genotypes §	8	High	Limited access to improved indigenous breeding stock	Existing farmer knowledge, low cost, compatibility with scavenging systems	Moderate	(2, 10, 59, 128)
A4. Implement safe and sustainable feed and water systems	5	Moderate-high	Seasonal water scarcity, feed availability, competing land uses	Use of local feed resources, simple environmental management practices, extension support, prevent wild bird access to food and water	Moderate-high	(42, 58, 59, 82)
B1. Build and sustain trust between veterinary services and family poultry producers	1, 2	Moderate	Historical mistrust, weak veterinary presence, inadequate compensation mechanisms	Long-term engagement, transparent governance, reliable service delivery	Very high	(7, 35, 77–79, 83, 86, 120, 130, 135)
B2. Integrate local knowledge systems into veterinary practice	3	High	Institutional resistance, limited training in participatory approaches	Relatively low cost, incorporation into veterinary curricula and extension programs	Moderate	(7, 87–89, 131, 132)
B3. Co-develop communication strategies with family poultry producers	7	High	Need for skilled facilitation and ongoing dialogue	Existing community networks, local languages, trusted intermediaries	High	(83, 89, 92, 94–97, 133, 134)
B4. Involve family poultry producers in technical solution development	6	Moderate-high	Additional time and facilitation requirements, donor preference for rapid implementation	Improved uptake, stronger local ownership, reduced intervention failure	High	(5, 18, 42, 54, 92–94, 102)
B5. Pre-negotiate outbreak preparedness protocols with communities	7	Moderate	Political sensitivity surrounding culling and compensation, coordination challenges	Existing local governance structures, preparedness planning processes	High	(22, 54, 79, 83, 84)
C1. Embed gender analysis in program design and monitoring	6, 9	Moderate-high	Limited gender expertise, weak collection of gender-disaggregated data	Increasing donor expectations, availability of gender-analysis tools	High	(17, 18, 92, 100–103, 119, 121, 123, 124)
C2. Ensure compensation and incentive schemes are gender-equitable	6, 9	Moderate	Legal, financial and administrative barriers; entrenched gender norms	Policy reform, digital payments, community verification mechanisms	High	(83, 84, 101, 103, 104, 119)
C3. Improve gender balance in animal health services	6, 9	Low-moderate	Long timeframes, educational barriers, recruitment and retention challenges	Scholarships, targeted recruitment, supportive workplace policies	Moderate-high	(80, 81, 101–103, 119, 129)
C4. Target women specifically in training and capacity-building programs	5, 6, 9	High	Women’s time constraints, literacy barriers, mobility restrictions in some settings	Women’s groups, NGOs, community-based training approaches	High	(18, 54, 92, 94, 102, 103, 105, 119, 129)
Overall package of recommendations	1–9	Moderate	Requires sustained political commitment, intersectoral coordination, and long-term investment	Integration with existing livestock development, One Health, and rural livelihood programs	Moderate-high	(5, 9, 10, 54, 59, 92, 107, 108, 112, 118)

* Feasibility and expected impact ratings were derived through qualitative assessment by the authors, informed by evidence identified in the literature review. Feasibility was assessed based on technical complexity, cost, infrastructure requirements, institutional capacity, and likelihood of adoption in resource-limited settings. Expected impact was assessed based on the reported contribution of each intervention to avian influenza prevention, detection, reporting, control, and broader poultry health system strengthening. Ratings represent comparative judgments rather than quantitative estimates and are intended to support prioritization and discussion. § Indicates the recommendation not relevant to LPAI.

Based on the evidence reviewed and the conceptual framework proposed here, we recommend adopting a systems approach by prioritizing control of ND, fowl cholera, and IBD, i.e., the important differential diagnoses for HPAI in field settings (22, 54, 75). Deploy community-based vaccination programs, wherever possible using thermotolerant vaccines capable of reaching remote household flocks (88). Ensure vaccine delivery systems address cold-chain constraints and that cross-sector collaboration supports cold-chain training, maintenance, and joint monitoring of vaccination coverage (85).

Reducing baseline mortality is both a productivity objective and a prerequisite for effective HPAI surveillance. Early-warning systems for HPAI depend on the ability to detect mortality levels that exceed expected background losses; where routine mortality is high or highly variable, unusual mortality events may be obscured, delaying disease recognition and reporting (106). Therefore, surveillance programs should combine serological and laboratory-based approaches with participatory epidemiology to improve understanding of local disease patterns, outbreak seasonality, and community priorities (71). Participatory approaches can enhance surveillance sensitivity, timeliness, and trust between communities and animal health services, although they require sustained investment and may be challenging to scale rapidly during emergency responses (120, 130).

Where LPAI is endemic or regularly detected, integrating surveillance into existing endemic poultry disease programs may improve efficiency, strengthen community engagement and enhance early detection, particularly when combined with participatory epidemiology and laboratory-supported surveillance (59, 62, 71). Participatory epidemiology may help identify subtle changes in flock productivity and health, although it should complement rather than replace laboratory-supported surveillance because reporting can be influenced by local incentives and perceptions of disease control measures (86, 120). Operationally, surveillance objectives differ between HPAI and LPAI. HPAI surveillance prioritizes rapid detection and response to outbreaks causing clinical disease, whereas LPAI surveillance focuses on detecting silent viral circulation, monitoring H5 and H7 subtypes, and identifying conditions that may favor viral evolution (20, 21, 23, 28).

Reporting systems should build on the intrinsic motivations of communities, including women, rather than relying primarily on financial incentives, as monetary rewards may increase short-term participation while reducing the sustainability of engagement once incentives are withdrawn (83, 122). Monitoring the participation of women and men in decision-making, training and communication activities is essential (121, 123, 124). Policy reforms should support community-based animal health worker programs, gender-disaggregated surveillance data collection, and value chain interventions that recognize gendered roles in poultry production and marketing (102).

2 Strengthen poultry value chain quality assurance

Enable reliable, safe, and equitable access to poultry markets. Co-design market infrastructure with poultry traders, consumers, and market managers to deliver sound hygiene and sanitation, disease surveillance, and equitable trading practices (22, 42, 125, 126).

3 Select locally adapted poultry genotypes

Encourage poultry genotypes adapted to local agroecological conditions. Indigenous and other locally adapted genotypes are generally more resilient under endemic disease pressure and resource-constrained production systems, remaining productive with fewer external inputs than highly selected commercial lines (40, 127, 128). Lower routine mortality and improved flock health facilitate recognition of unusual mortality events, which are central to early detection and reporting of HPAI incursions (42).

4 Implement safe and sustainable feed and water systems

Feed entering poultry-producing areas should be free from contamination with poultry pathogens, including AIVs (42). Encourage feed systems that reduce food–feed competition by utilizing feedstuffs unsuitable for direct human consumption, while ensuring nutritional adequacy and feed safety (82). Safe feed is a foundational biosecurity measure, particularly relevant where high proportions of households keep poultry and where formal biosecurity extension is limited.

Environmental management is critical for LPAI control in scavenging systems. Practical measures include protecting feed and water from wild birds, improving drainage, and managing flock congregation points (58, 59).

B Addressing governance, institutional and social barriers

1 Build and sustain trust between veterinary services and family poultry producers

We propose that government agencies invest in veterinary extension systems that are specifically resourced to engage with family poultry producers (2, 10), not only with large livestock or commercial poultry enterprises. Stronger links between services and producers are associated with better reporting, earlier detection, and greater voluntary compliance with biosecurity measures. Trust (7, 77−79, 135) is earned through consistent, respectful engagement, the honoring of compensation commitments, and visible follow-through on community concerns. Where uncompensated culling has occurred in the past, proactive trust-repair strategies, including community-level dialogue and transparent accountability mechanisms, are essential preconditions for effective future response.

2 Integrate local knowledge systems into veterinary practice

Veterinary services should build the capacity to understand and work respectfully with local knowledge systems, including ethnoveterinary beliefs and community explanations of disease (131, 132). In many settings, livestock keepers draw on both traditional and biomedical sources of animal healthcare. Training curricula and extension programs should therefore prepare animal health workers to navigate the cross-cultural dimensions of animal health practice (88).

3 Co-develop communication strategies with family poultry producers

Risk communication should shift toward participatory communication grounded in two-way dialogue, local languages, and community ownership (92, 94, 133). Communication strategies must be co-developed with family poultry producers of all genders and with specialists familiar with family poultry systems. Messages should distinguish between households that keep poultry primarily as assets and those that depend on poultry for cash income (134). Strategies should incorporate feedback loops so that messages are continuously refined in response to community experience (83).

4 Involve family poultry producers in technical solution development

Family poultry producers, particularly women, should be active participants in the co-design of technical solutions (17, 18, 54), not merely recipients of interventions developed elsewhere. Context-appropriate solutions, without imposing costs that are prohibitive at the household scale, are more likely to be sustained than wholesale adoption of models developed for intensive systems.

5 Pre-negotiate outbreak preparedness protocols with communities

Negotiate outbreak response protocols, particularly for HPAI preparedness, including detection, reporting, movement controls, culling, and compensation arrangements, with local communities before outbreaks occur (54). Pre-negotiation builds the relational foundation for rapid voluntary compliance during an emergency and avoids the trust-destroying experience of communities encountering untransparent, uncompensated culling for the first time in a crisis. Include culturally appropriate compensation and incentive schemes, trained community animal health workers, and links to local government representatives.

C Addressing gender and socio-economic inequities

1 Embed gender analysis in program design and monitoring

National and international agencies should require systematic gender analysis of HPAI control programs, including gender-disaggregated data collection in research, surveillance, and monitoring systems (102). Planning and technical teams should move beyond tokenistic or consultative participation by ensuring that women and women-focused organizations have meaningful influence over priorities, program design, resource allocation, implementation, and evaluation. Program design should strengthen women’s agency and decision-making power by addressing their differential access to vaccines, training, information, financial resources, compensation mechanisms, and decision-making forums, rather than simply increasing their representation within existing structures. Program monitoring should therefore assess not only the number of women and men participating in community-based trainings, HPAI communication campaigns, and decision-making bodies, but also whether women have meaningful opportunities to influence decisions and whether their priorities are reflected in resulting actions.

2 Ensure compensation and incentive schemes are gender-equitable

Fair compensation policies must recognize that poultry are frequently owned and managed by women and children in family systems (17, 83). Compensation and recovery measures should account for the gendered distribution of poultry ownership, labor and income, and should avoid reinforcing existing inequalities by directing compensation solely through male household heads or through systems that favor larger, commercially managed poultry enterprises (17). Where eligibility or payment procedures rely on forms of ownership, registration or financial access that may exclude women, alternative mechanisms should be established to ensure that women who own, manage or depend economically on poultry can directly access compensation. Compensation schemes should also distinguish between family and commercial producers, recognizing that measures designed primarily around commercial production may disadvantage women involved in small-scale poultry production (17).

3 Improve gender balance in animal health services

National and international agencies should take deliberate measures to improve gender balance across animal health systems, from field veterinarians and extension personnel to village livestock agents and community animal health workers. This should include targeted recruitment, training and retention of women, particularly in frontline roles where animal health workers are often the primary interface with women livestock and poultry keepers (17, 81, 103). In some settings, women farmers are more likely to seek services from, and share information with, female animal health workers. Increasing the representation of women in animal health services is therefore not only an equity imperative but a practical strategy for improving surveillance sensitivity and intervention uptake in communities where women manage family poultry.

4 Target women specifically in training and capacity-building programs

Programs should explicitly prioritize women’s participation in training on poultry care and avian influenza control, rather than including women as an afterthought in generic farmer training (17, 18, 54, 129). Building the capacity of women’s organizations and NGOs enhances their ability to disseminate information to rural households, specifically reaching women with limited mobility or limited formal schooling. This strengthens both the reach of communication and local ownership of disease control outcomes.

### Limitations

This review has several limitations that should be considered when interpreting its findings and conclusions. As a narrative, policy-oriented rapid review, the search strategy was structured but non-exhaustive, and relevant evidence may therefore have been missed. Study screening, eligibility assessment and data extraction were undertaken by one author to meet the timeframe and pragmatic requirements of a rapid review; although predefined eligibility criteria were applied, the absence of independent, dual screening and data extraction may have increased the risk of selection or extraction errors. The review incorporated policy and guidance documents alongside peer-reviewed literature, reflecting its policy-oriented purpose but limiting direct comparison between evidence types. The thematic synthesis, conceptual framework and recommendations necessarily involved author interpretation, and the feasibility and impact ratings in Table 3 represent qualitative judgments rather than systematic or quantitative evaluations. Furthermore, the predominantly observational and narrative nature of the evidence limits causal inference. The proposed interventions and their potential effects on avian influenza prevention and control therefore require systematic empirical evaluation, including assessment of their cost-effectiveness and potential effects on reporting and response times. Further research is also needed to understand how biological and ecological hazards interact with exposure pathways, structural and socio-economic vulnerabilities, and institutional capacities within family poultry systems.

## Conclusion

Drawing together the evidence reviewed and the conceptual synthesis developed in this paper, we conclude the following. Controlling HPAI and LPAI in family poultry production systems requires more than biosecurity protocols; it also requires systemic transformation of poultry health and production systems grounded in trust, community engagement, and cross-sectoral collaboration. Strengthening disease prevention while simultaneously supporting food security, livelihoods, and resilience is not only possible, it is essential.

The three-part package of recommendations proposed (spanning structural constraints within family poultry systems; governance, institutional and social barriers; and gender and socio-economic equity) addresses the nine factors identified in Table 2 as relevant to control feasibility. The feasibility and expected impact of these recommendations are summarized in Table 3. None of these measures is sufficient in isolation: it is their coordinated implementation, as depicted in the conceptual systems framework in Figure 1, that determines whether control efforts translate into sustained reductions in disease risk. In our view, poultry systems can be made more resilient to future shocks and more supportive of sustainable development by embedding HPAI and LPAI control into a One Health, multi-hazard risk management framework that reflects real-world complexity. Realizing this vision will require sustained political commitment and coordinated investment from national veterinary and public health services, regional and international bodies including the Quadripartite (FAO, WHO, WOAH and UNEP), and donors, working in genuine partnership with family poultry producers themselves.
