# Supplementary material for: Rethinking avian influenza control: a systems-oriented One Health perspective for family poultry
Source: Front Vet Sci. 2026 Oct 19;13:1814751. doi: 10.3389/fvets.2026.1814751 (PMC13725106; doi:10.3389/fvets.2026.1814751)
Supplement: Supplementary file 1 [file Table_1.docx]

**Supplementary Table S1.** Search strategy, databases searched, search strings, and study selection summary.

| Search No. | Database search string | PubMed number identified | Web of Science number identified | Duplicates | Number of titles & abstracts screened | Number of full reviews | Number of full-text articles excluded |
| --- | --- | --- | --- | --- | --- | --- | --- |
| 1 | "avian influenza" AND ("family poultry" OR "small-scale poultry" OR "backyard poultry" OR "village poultry" OR "smallholder poultry") | 203 | 297 | 89 | 211 | 63 | 40 |
| 2 | "avian influenza" AND "One Health" AND ("family poultry" OR "small-scale poultry" OR "backyard poultry" OR "village poultry" OR "smallholder poultry") | 10 | 11 | 6 | 15 | 12 | 0 |
| 3 | "avian influenza" AND "One Health" AND “gender” AND ("family poultry" OR "small-scale poultry" OR "backyard poultry" OR "village poultry" OR "smallholder poultry") | 0 | 0 | - | - | - | - |
| 4 | "avian influenza" AND "One Health" AND “gender” | 3 | 5 | 2 | 6 | 6 | 0 |
| 5 | "avian influenza" AND "One Health" AND (“socioeconomic” OR “socio-economic”) | 27 | 26 | 15 | 20 | 11 | 9 |
| 6 | "avian influenza" AND “biosecurity” AND ("family poultry" OR "small-scale poultry" OR "backyard poultry" OR "village poultry" OR "smallholder poultry") | 60 | 94 | 53 | 56 | 26 | 15 |
| 7 | "indigenous poultry" AND "disease tolerance" | 0 | 0 | - | - | - | - |
| 8 | "disease resistance" AND ("family poultry" OR "small-scale poultry" OR "backyard poultry" OR "village poultry" OR "smallholder poultry") | 1 | 5 | 1 | 5 | 3 | 0 |
| 9 | “gender” AND ("family poultry" OR "small-scale poultry" OR "backyard poultry" OR "village poultry" OR "smallholder poultry") | 16 | 35 | 14 | 30 | 29 | 13 |
| 10 | ("family poultry" OR "small-scale poultry" OR "backyard poultry" OR "village poultry" OR "smallholder poultry") AND “gender” AND (“socioeconomic” OR “socio-economic”) | 2 | 2 | 0 | 4 | 4 | 1 |
| 11 | ("family poultry" OR "small-scale poultry" OR "backyard poultry" OR "village poultry" OR "smallholder poultry") AND "One Health" | 55 | 45 | 33 | 37 | 30 | 15 |
| 12 | "avian influenza" AND "One Health" AND ("cost-benefit analysis" OR "cost benefit analysis") | 2 | 0 | - | 2 | 2 | 0 |
| 13 | "One Health" AND ("cost-benefit analysis" OR "cost benefit analysis") | 99 | 13 | 1 | 33 | 4 | 2 |
| 14 | "gender framework" AND “poultry” | 0 | 0 | - | - | - | - |
| 15 | "gender framework" AND "livestock systems" | 1 | 1 | 1 | 1 | 1 | 0 |
| 16 | "avian influenza" AND “biosecurity” AND “livelihood” | 4 | 7 | 3 | 6 | 4 | 0 |
| 17 | "avian influenza" AND ("risk communication" OR communication) AND ("family poultry" OR "small-scale poultry" OR "backyard poultry" OR "village poultry" OR "smallholder poultry") | 7 | 9 | 3 | 7 | 6 | 2 |
| 18 | "avian influenza" AND "systems thinking" | 1 | 1 | 1 | 1 | 1 | 1 |
| 19 | "avian influenza" AND ("food security" OR “nutrition”) AND ("family poultry" OR "small-scale poultry" OR "backyard poultry" OR "village poultry" OR "smallholder poultry") | 15 | 29 | 13 | 25 | 20 | 14 |
| 20 | "One Health" AND ("multi-hazard risk reduction" OR "multi-hazard management" OR "multi-hazard") | 4 | 3 | 1 | 6 | 6 | 6 |
|  | TOTALS | 510 | 583 | 236 | 465 | 228 | 118 |

**Screening summary**

| **Stage** | **Number (n)** |
| --- | --- |
| Total records identified | 1093 |
| Duplicate records removed | 236 |
| Records screened (title and abstract) | 465 |
| Records excluded after title/abstract screening | 237 |
| Full-text articles assessed for eligibility | 228 |
| Full-text articles excluded | 118 |
| Peer-reviewed publications included | 110 |
| Policy and guidance documents included | 27 |
| Total references included in review | 137 |
